# Estrogen Receptor Beta-Mediated Modulation of Lung Cancer Cell Proliferation by 27-Hydroxycholesterol

**DOI:** 10.3389/fendo.2018.00470

**Published:** 2018-08-23

**Authors:** Shiro Hiramitsu, Tomonori Ishikawa, Wan-Ru Lee, Tamor Khan, Christine Crumbley, Nimra Khwaja, Faezeh Zamanian, Arvand Asghari, Mehmet Sen, Yang Zhang, John R. Hawse, John D. Minna, Michihisa Umetani

**Affiliations:** ^1^Center for Nuclear Receptors and Cell Signaling, University of Houston, Houston, TX, United States; ^2^Department of Biology and Biochemistry, College of Natural Sciences and Mathematics, University of Houston, Houston, TX, United States; ^3^Division of Pulmonary and Vascular Biology, Department of Pediatrics, University of Texas Southwestern Medical Center, Dallas, TX, United States; ^4^Department of Pharmacological and Pharmaceutical Sciences, College of Pharmacy, University of Houston, Houston, TX, United States; ^5^Department of Biochemistry and Molecular Biology, Mayo Clinic, Rochester, MN, United States; ^6^Hamon Center for Therapeutic Oncology Research, University of Texas Southwestern Medical Center, Dallas, TX, United States

**Keywords:** lung cancer, estrogen receptor, ERβ, 27-hydroxycholesterol, cholesterol metabolites

## Abstract

27-hydroxycholesterol (27HC) is an abundant cholesterol metabolite in human circulation and promotes breast cancer cell proliferation. Although lung is one of the organs that contain high levels of 27HC, the role of 27HC in lung is unknown. In this study, we found that 27HC promotes lung cancer cell proliferation in an estrogen receptor β (ERβ)-dependent manner. The expression of 27HC-generating enzyme CYP27A1 is higher in lung cancer cells than in normal lung cells. Treatment with 27HC increased cell proliferation in ERβ-positive lung cancer cells, but not in ERα-positive or ER-negative cells. The effect on cell proliferation is specific to 27HC and another oxysterol, 25-hydroxycholesterol that has a similar oxysterol structure with 27HC. Moreover, among ligands for nuclear receptors tested, only estrogen had the proliferative effect, and the effect by 27HC and estrogen was inhibited by ERβ-specific, but not ERα-specific, inhibitors. In addition, the effect by 27HC was not affected by membrane-bound estrogen receptor GPR30. Interestingly, despite the high expression of CYP27A1, endogenously produced 27HC was not the major contributor of the 27HC-induced cell proliferation. Using kinase inhibitors, we found that the effect by 27HC was mediated by the PI3K-Akt signaling pathway. These results suggest that 27HC promotes lung cancer cell proliferation via ERβ and PI3K-Akt signaling. Thus, lowering 27HC levels may lead to a novel approach for the treatment of lung cancer.

## Introduction

Lung cancer is the leading cause of cancer deaths in men and women in the United States (1), and there is accumulating evidence that estrogen is a major driver of lung cancer (2). In addition, the proportional occurrence of histological lung carcinoma subtypes differs between men and women. While squamous cell carcinoma is the most common subtype in men, adenocarcinoma is the most common subtype in women (1, 3). Estrogen receptors, ERα and ERβ, are members of the nuclear receptor family, and are present both in normal lung tissue and lung tumors with ERβ as a dominant isoform (4–6). Large-scale randomized and observational studies showed that women who used hormone replacement therapy (HRT) including estrogen have a higher risk of adenocarcinoma than women who did not use HRT, and that risk of lung cancer was associated with chronic use of HRT (7). In contrast, breast cancer patients who received anti-estrogen treatment had significantly lower subsequent lung cancer mortality (8). Studies using genetically modified mice showed that both male and female lungs are highly estrogen-responsive (9, 10). Mice deficient in ERβ exhibited significant lung dysfunction both in males and females, indicating the importance of ERβ in the maintenance of lung function (11). Estrogens also stimulate growth and progression of lung tumors, and local production of estrogen in lung tissues by aromatase could affect lung tumor progression in ER(+) malignancies in men and women (12, 13). ER also interacts with epidermal growth factor receptors (EGFRs), and the combination of EGFR tyrosine kinase inhibitors and ER antagonists gives maximal inhibition of tumor cell proliferation *in vitro* and also anti-tumor activity in mouse tumor xenograft models *in vivo* (14, 15). Taken together, estrogen and ERs play important roles in lung cancer pathogenesis and treatment.

Oxysterols are metabolites of cholesterol that are produced in the liver and other peripheral tissues as a means to eliminate cholesterol (16). The most abundant circulating oxysterol is 27-hydroxycholesterol (27HC), and serum concentrations of 27HC correlate well with that of cholesterol. The levels of 27HC also rise progressively with age. The enzyme that generates 27HC, sterol 27-hydroxylase (CYP27A1), is primarily expressed in the liver, but also in peripheral tissues to a lesser extent (17). Using cell-based and *in vitro* assays, we discovered that 27HC is a competitive ER antagonist in the cardiovascular system (18). We further found that 27HC binds directly to ERα (*K*_*i*_ = 1.32 μM) and ERβ (*K*_*i*_ = 0.42 μM) in their ligand binding pockets, and it inhibits both transcriptional and non-transcriptional estrogen-dependent production of nitric oxide by vascular cells. In mice, elevated 27HC levels decreased ER-dependent expression of vascular nitric oxide synthase and repressed carotid artery reendothelialization after vascular injury. In addition to the anti-estrogenic effects of 27HC in vascular cells, we identified pro-estrogenic actions of 27HC in hepatoma HepG2 and colon cancer Caco-2 cells (18). Combinatorial peptide phage display revealed that 27HC induces a unique active conformation of ERα (19). In contrast to estrogens that have various levels of agonistic activity in all tissues, selective ER modulators (SERMs) are compounds that act as agonists or antagonists depending on the target genes and tissues (16). Although many compounds have been identified as SERMs, all of them were synthetic compounds. Thus, 27HC is the first identified endogenously produced SERM, and has important biological actions *in vitro* and *in vivo*.

In breast cancer, in which ERα is classically involved in the development and progression of many tumors, paradoxically, post-menopausal women with decreased estrogen production are particularly at increased risk of ER-positive breast cancer. This risk occurs at a time when circulating estrogen levels are declining, and endocrine-based therapies against ER-positive breast cancer employing synthetic SERMs or aromatase inhibition are often ineffective or acquire resistance, suggesting other important unknown ER-mediated mechanisms (20). Once we identified 27HC as a novel endogenous SERM and that its abundance increases with age, we tested its potential actions on ER-positive breast cancer cells, and found that 27HC up-regulated ERα target gene expression and increased cell proliferation (19). Also in mice, 27HC promoted tumor growth and metastasis to the lung in an orthotropic breast tumor xenograft model (21). In humans, we found that elevated levels of 27HC in tumors, which are correlated with reduced expression of the 27HC-catabolizing enzyme oxysterol 7α-hydroxylase (CYP7B1), are associated with poorer patient survival (22). Even when age, tumor size, nodal status, and perioperative therapy are taken into consideration, low expression of *CYP7B1* continues to be associated with poor overall outcome. Thus, 27HC is a non-estrogen, locally-modulated, non-aromatized ER ligand that stimulates ER-positive breast tumor growth, and, most importantly, it is abundant in the microenvironment of tumors in women.

In the present study, we investigated how 27HC impacts lung cancer cell proliferation through its modulation of the ER-mediated action. We found that ERβ expression is higher in lung cancer cells than in normal lung cells, and also that 27HC promotes ERβ (+) lung cancer cell proliferation. Although lung cancer cells have elevated gene expression of 27HC-producing enzyme CYP27A1, endogenously produced 27HC was not the major factor involved in the 27HC-induced cell proliferation. We sought to determine the underlying mechanism, and found that the PI3K-Akt pathway is involved in the effect by 27HC on lung cancer cell proliferation.

## Materials and methods

### Materials

27HC was purchased from Avanti Polar Lipids. T0901317 (T1317) was purchased from Cayman Chemical. 1,3-bis(4-hydroxyphenyl)-4-methyl-5-[4-(2-piperidinylethoxy)phenol]-1H-pyrazole dihydrochloride (MPP), 4-[2-phenyl-5,7-bis(trifluoromethyl) pyrazolo[1,5-a]pyrimidin-3-yl]phenol (PHTPP), G1, G15, and iressa were purchased from Tocris Bioscience. Cholestane-3β,5α,6β-triol and 5α-hydroxy-6-ketocholesterol were purchased from Steraloids. 17β-estradiol (E_2_), GW3965 (GW), 4β-hydroxycholesterol, 7-ketocholesterol, 22(R)-hydroxycholesterol, 24(S)-hydroxycholesterol, 25-hydroxycholesterol, cholesterol 5α,6α-epoxide, cholesterol 5β,6β-epoxide, cholesterol, progesterone, 5α-dihydrotestosterone (DHT), dexamethasone, cortisone, Wy-14643, GW501516, troglitazone, EGF, insulin-like growth factor (IGF), vascular endothelial growth factor (VEGF), PD0325901, U0126, SB203580, LY294002, and clotrimazole were purchased from Sigma-Aldrich.

### Gene expression analyses and assessments of gene expression

Expression profiling of *ER*α, *ER*β, *CYP27A1*, and *CYP7B1* was part of a larger study (Gene Expression Omnibus DataSets accession number GSE32036) that has been previously published (23, 24). Raw data was background corrected with RMA, Log2 transformed, and summarized by medianpolish. Differently expressed genes were called using the LIMMA package and *p*-values were corrected for multiple testing with the Benjamini-Hochberg procedure. The expression heatmap was generated in R and ontology enrichment was performed in HOMER. *CYP27A1, CYP7B1, ER*α, and *ER*β transcript abundance were further evaluated by quantitative RT-qPCR (25). Primer sequences used for RT-qPCR are listed in Supplemental Table 1.

### Cell culture assays

MCF-7 (ATCC), A549 (ATCC), Lewis lung carcinoma (LLC, ATCC), H1395, and H596 cells were maintained in RPMI 1640 medium containing 5% FBS. Cell proliferation was assessed by quantifying ^3^H-thymidine incorporation (22). Cells were maintained in phenol red-free DMEM containing 5% charcoal-stripped FBS for 72 h. Cells were then passed into 24-well plates at a density of 100,000 cells per ml in media containing 5% charcoal-stripped FBS, and 24 h later they were treated with compounds for 24 h, during which ^3^H-thymidine incorporation was quantified. To provide a complementary approach in H1395 cells, in selected studies cell proliferation was quantified by cell number using FluoReporter Blue Fluorometric dsDNA Quantitation kit (Thermo Fisher Scientific). To evaluate the involvement of ERβ in the 27HC-induced lung cancer cell proliferation, LLC cells were transfected with an ERβ-expression plasmid using Lipofectamine 2000 and used for the cell proliferation assay. To specifically evaluate the impact of silencing of *CYP27A1* or *CYP7B1* on the modulation of lung cancer cell proliferation, their expression was knocked down using dsRNA targeting human *CYP27A1* (TF313602, OliGene Technologies), *CYP7B1* (Dharmacon), or control dsRNA. H1395 cells were transfected with 50 nM RNA as described previously (25) and cell proliferation responses to vehicle or 27HC were evaluated from 48 to 72 h post-transfection.

### Immunoblot analyses

ER protein abundance was assessed by immunoblot analysis using antibodies against ERα (ab75635, Abcam), ERβ (26), and GAPDH (G8796, Sigma-Aldrich) as a control. Phosphorylation of ERK1/2, p38 MAPK, and Akt proteins were assessed by immunoblot with their phosphorylated protein-specific antibodies (Cell Signaling), and their total protein abundance was also assessed.

### Statistical analysis

All data are expressed as mean ± SEM. Two-tailed Student's *t*-test or ANOVA was used to assess differences between two groups or among more than two groups, respectively, with Newman-Keuls *post-hoc* testing following ANOVA. *P*-values < 0.05 were considered significant.

## Results

### Gene expression of ER in lung cancer cell lines

**Figure 1:**
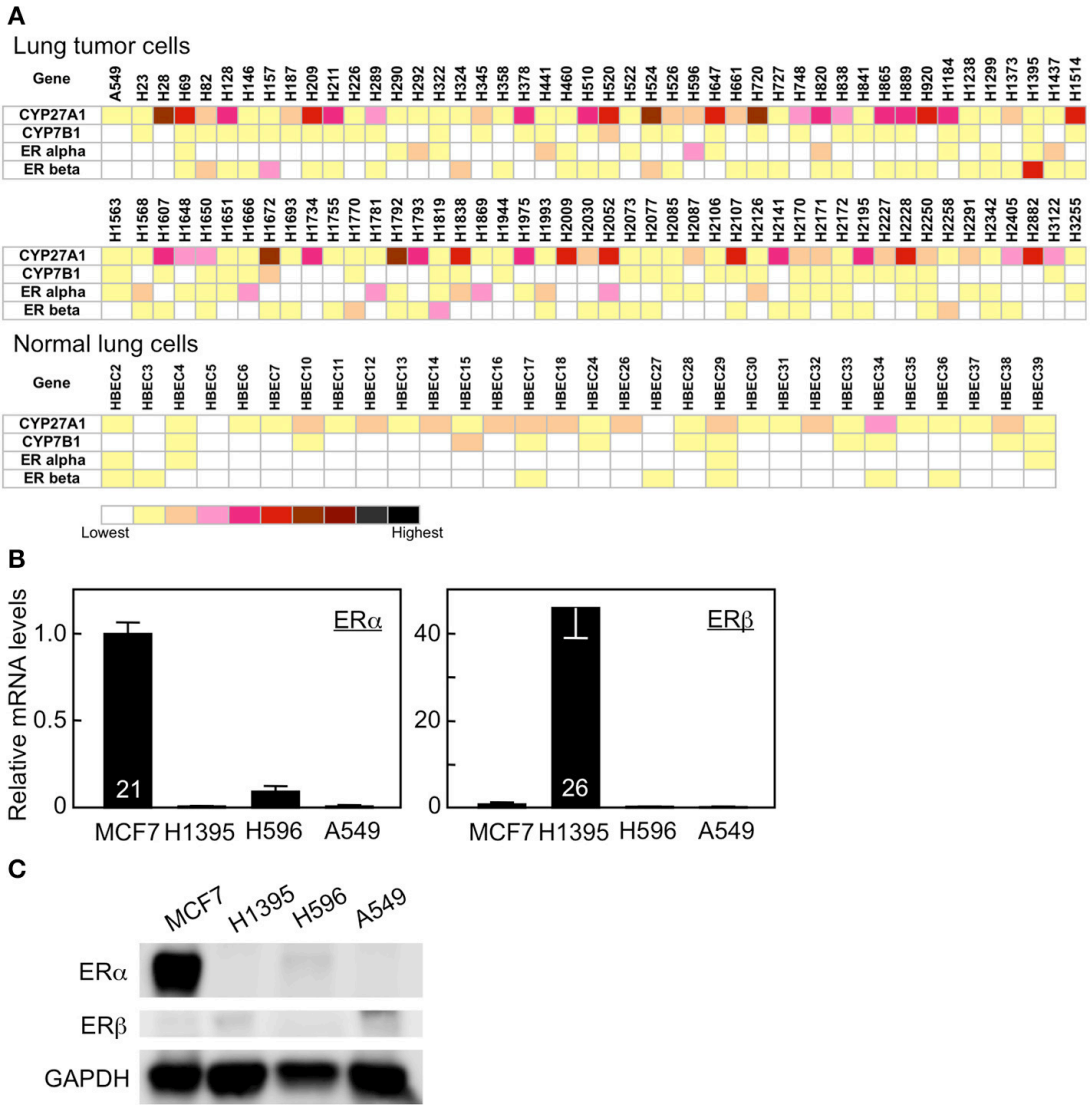
Gene expression in human lung tumor and normal bronchial epithelial cells. **(A)** Gene expression profile of *CYP27A1, CYP7B1, ER*α, and *ER*β. **(B)** qRT-PCR analysis of *ER*α, and *ER*β in MCF7, H1395, H596, and A549 cancer cells (*n* = 6–7). Cycle time of the highest expressing group for each gene is indicated inside its corresponding bar. **(C)** Immunoblots of ERα, ERβ, and GAPDH proteins.

To evaluate possible actions of 27HC in lung cancer pathogenesis, we determined the expression levels of *CYP27A1, CYP7B1, ER*α, and *ER*β in a variety of lung cancer and normal lung cell lines. Ninety-one human lung tumor cell lines and 30 human normal bronchial epithelial cell lines from the Lung Cancer Program at UT Southwestern Medical Center were analyzed. With relative levels of expression represented as log_2_ (signal) in the heat diagram, mRNAs for *CYP27A1* were detectable at varying levels with clear difference among the different cell types (Figure 1A). Thirty-one tumor cell lines showed moderate to high expression of 27HC-generating enzyme CYP27A1 while none of the normal cell line showed moderate to high *CYP27A1* expression. The log_2_ *CYP27A1* expression ratio of tumor vs. normal lung cells is 0.87 (*P* < 0.001). The mRNA expression for *ER*β was also higher in tumor cells than normal lung cells (log_2_ ratio 0.28, *P* = 0.004). There were also varying levels of mRNA expression for *CYP7B1* and *ER*α transcripts, although there is no significant difference between tumor vs. normal lung cells. We further validated the microarray data and also compared the mRNA expression levels in high ERβ expressed H1395, high ERα expressed H596, and ER-negative A549 cells with those in breast cancer MCF-7 cells by qRT-PCR analysis. As expected, MCF-7 cells have high mRNA expression of *ER*α. Consistent with the microarray results (Figure 1A), H1395 and H596 cells have high expression of *ER*β and *ER*α, respectively (Figure 1B). The protein expression of ERα and ERβ was also confirmed in MCF-7 and H1395 cells, respectively (Figure 1C). The mRNA expression of *CYP27A1* and *CYP7B1* varied among cell lines, and the gene expression of aromatase, which converts testosterone to estrogen, was not observed in the cells tested (Supplemental Figure 1 and data not shown). These results suggest that the molecular machinery necessary to synthesize and metabolize 27HC is found in lung cancer cells, and local 27HC production and *ER*β mRNA expression are higher in lung tumor cells than normal lung cells.

### 27HC promotes H1395 lung cancer cell proliferation

**Figure 2:**
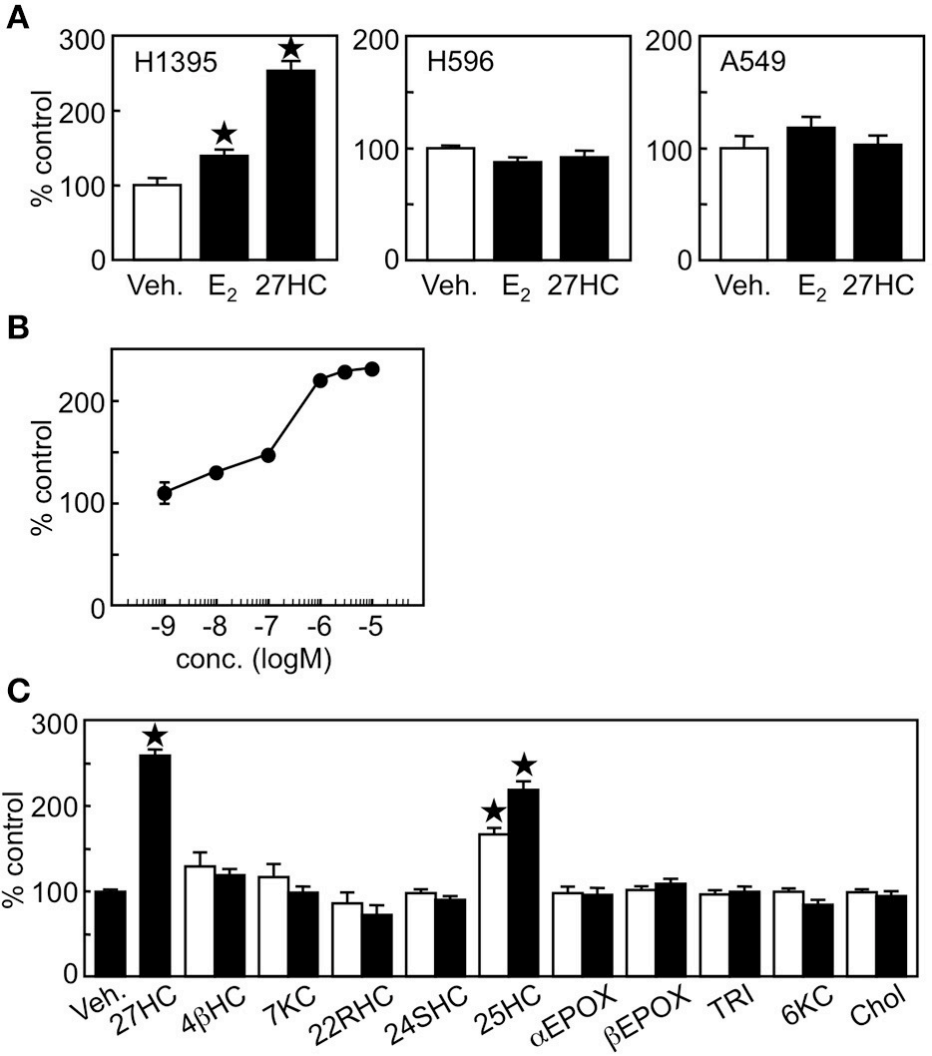
27HC promotes H1395 lung cancer cell proliferation. **(A)** Cell proliferation assay. Vehicle control, E_2_ (10 nM), or 27HC (1 μM) were treated for 24 h (*n* = 4). **(B)** Dose response of 27HC in H1395 cell proliferation (*n* = 4). **(C)** H1395 cell proliferation in the presence of oxysterols for 24 h (*n* = 4). 4βHC, 4β-hydroxycholesterol; 7KC, 7keto-cholesterol; 22RHC, 22(R)-hydroxycholesterol; 24SHC, 24(S)-hydroxycholesterol; 25HC, 25-hydroxycholesterol; αEPOX, cholesterol 5α,6α-epoxide; βEPOX, cholesterol 5β,6β-epoxide; TRI, cholestane-3β,5α,6β-triol; 6KC, 5α-hydroxy-6-ketocholesterol; and Chol, cholesterol at 0.1 μM (open bars) and 1 μM (closed bars). ^*^p < 0.05 vs. vehicle control.

Next, we evaluated whether 27HC has cell proliferative effects in lung cancer cells. We selected H1395 and H596 lung adenocarcinoma cells, which express high levels of *ER*β and *ER*α, respectively and also A549 cells which have no expression of ER (Figure 1). 27HC and E_2_ induced cell proliferation of H1395, but not H596 or A549 cells (Figure 2A), suggesting that the effect by 27HC is ERβ-dependent. The proliferative effects of 27HC are also dose-dependent (Figure 2B) and specific to the 27HC structure, but not to general oxysterols. Among other oxysterols tested, only 27HC, and 25-hydroxycholesterol were able to induce cell proliferation in this model (Figure 2C). 25-Hydroxycholesterol has a similar oxysterol structure as 27HC and also acts as an ER ligand. We could not see any promoting effect on the cell proliferation by 4β-Hydroxycholesterol, 7-keto-cholesterol, 22(R)-hydroxycholesterol, 24(S)-hydroxycholesterol, cholesterol 5α,6α-epoxide, cholesterol 5β,6β-epoxide, cholestane-3β,5α,6β-triol, and 5α-hydroxy-6-ketocholesterol, which are reported to stimulate breast cancer cell proliferation (27). These results indicate that 27HC promotes H1395 lung cancer cell proliferation and that the effect is specific to 27HC and 25-hydroxycholesterol.

### Effects of 27HC on lung cancer cell proliferation are ERβ-specific

**Figure 3:**
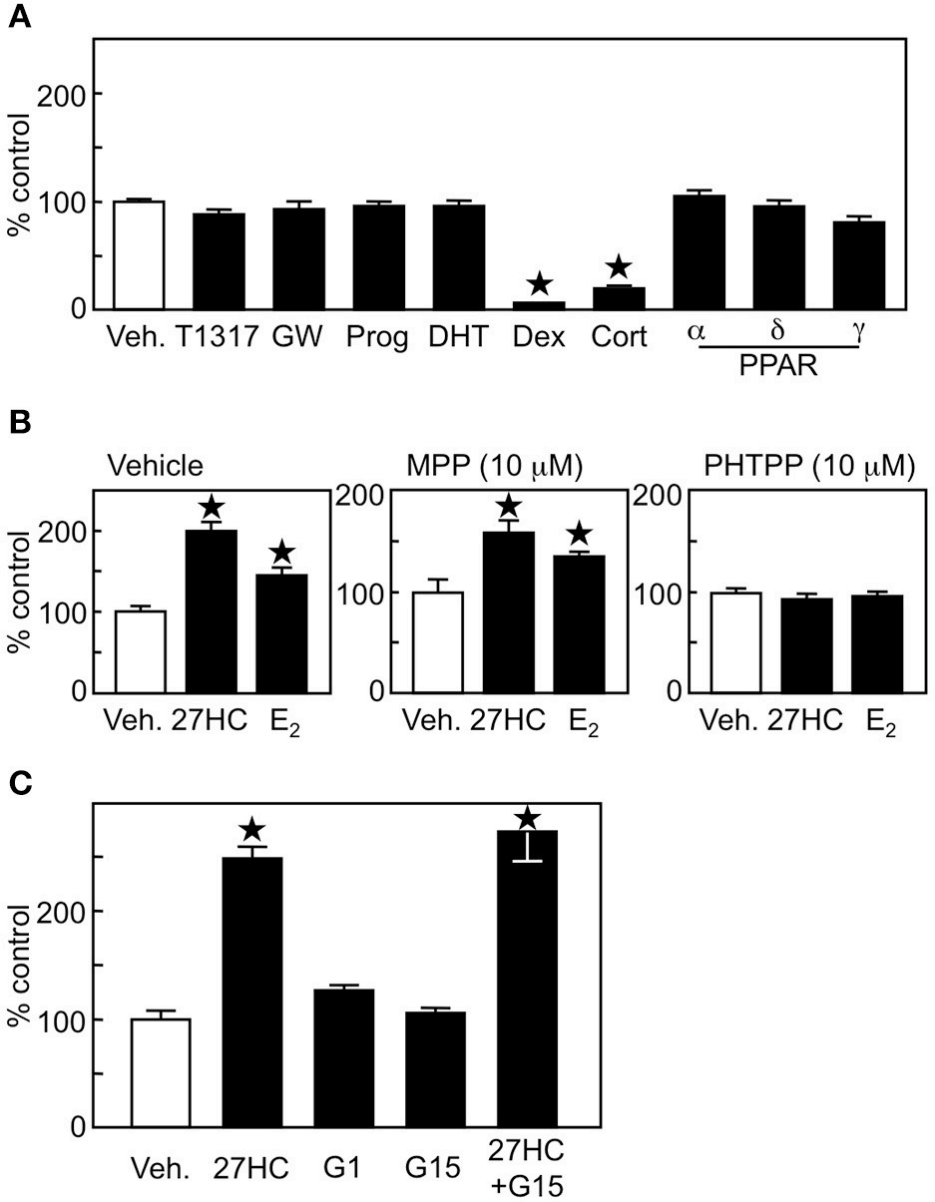
27HC promotes H1395 cell proliferation in an ERβ-dependent manner. **(A)** Cell proliferation assay of H1395 cells was performed using ligands for nuclear receptors (*n* = 4). T1317 (1 μM), GW (1 μM), Prog, progesterone (100 nM); Dex, dexamethasone (10 nM); Cort; cortisone (10 nM); α, Wy-14643 (1 μM); δ, GW501516 (1 μM); and γ, troglitazone (1 μM). **(B)** Effect of ER isoform-specific antagonist on the 27HC-induced H1395 cell proliferation (*n* = 4). **(C)** Effect of GPR30 on the cell proliferation induced by 27HC. H1395 cells were treated with vehicle, 27HC (1 μM), G1 (100 nM), G15 (100 nM), or 27HC plus G15 for 24 h (*n* = 4). ^*^p < 0.05 vs. vehicle control.

To examine whether the effect of 27HC on lung cancer cell proliferation was ERβ-specific, ligands for other nuclear receptors were tested on H1395 cells. Ligands for liver X receptor (LXR, T1317, GW), progesterone receptor (progesterone), androgen receptor (5α-dihydrotestosterone), glucocorticoid receptor (dexamethasone), mineralocorticoid receptor (cortisone), or α (Wy-14643), δ (GW501516), and γ (troglitazone) isoforms of PPAR in the concentrations known to activate their receptors (18) did not induce growth promoting effects in H1395 cells (Figure 3A). Together with the results in Figure 2C that 22(R)-hydroxycholesterol and 24(S)-hydroxycholesterol, which also act as ligands for LXR and retinoic acid related orphan receptors (RORs), respectively (28, 29), did not show any promoting effect on the H1395 cell proliferation, these results indicate that the effect by 27HC is not LXR-, PR-, AR-, MR-, GR-, PPAR-, or ROR-dependent. Next, we used ER isoform-specific inhibitors. The effect by 27HC and E_2_ was inhibited by the ERβ-specific inhibitor PHTPP, but not by the ERα-specific inhibitor MPP (Figure 3B). To confirm the importance of ERβ in the 27HC-mediated lung cancer cell proliferation, we compared the effect by 27HC on LLC cells, which are ER-negative, and LLC cells that overexpress ERβ following transfection with an ERβ expression plasmid. 27HC and E_2_ induced cell proliferation of LLC cells that expressed ERβ, but had no effect on parental LLC cells (Supplemental Figure 2), further indicating that the effect by 27HC on lung cancer cell proliferation is ERβ-dependent. In addition to classical ER, estrogen can also activate the G protein-coupled receptor GPR30 (6). Thus, the involvement of GPR30 in the H1395 cell proliferation was tested using its agonist G1 and antagonist G15. Neither of these GPR30 ligands affected H1395 cell proliferation and G15 did not abrogate the inducing effects of 27HC (Figure 3C), indicating that GPR30 is not involved in the 27HC-induced H1395 cell proliferation. These results indicate that 27HC promotes lung cancer cell proliferation via ERβ.

### Exogenous 27HC is the major effector on ERβ (+) lung cancer cell proliferation

**Figure 4:**
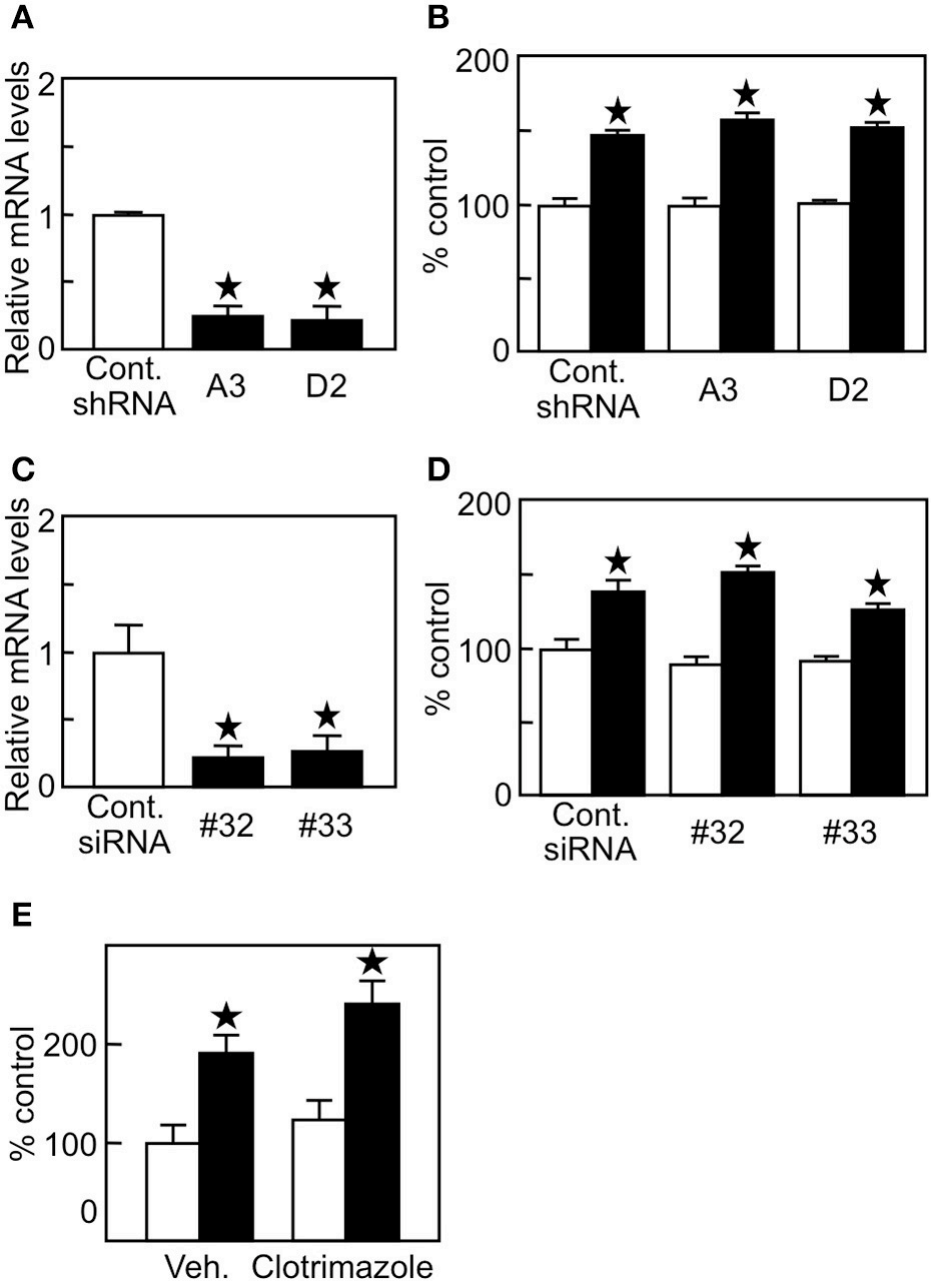
Endogenous production of 27HC is not a major contributor in H1395 cell proliferation. **(A)** *CYP27A1* gene expression (*n* = 4). **(B)** Cell proliferation assay using H1395 cells with shRNA against *CYP27A1* (*n* = 4). Open bars, vehicle treatments; closed bars, 27HC (1 μM) treatments. **(C)** *CYP7B1* gene expression (*n* = 4). **(D)**. cell proliferation assay using H1395 cells with siRNA against *CYP7B1* (*n* = 4). Open bars, vehicle treatments; closed bars, 27HC (1 μM) treatments. ^*^p < 0.05 against vehicle control. **(E)** Effect by clotrimazole on the 27HC-induced cell proliferation. Cells were treated with vehicle or 27HC (1 μM) in the absence (Veh.) or presence of clotrimazole (1 μM) for 24 h (*n* = 4). ^*^p < 0.05 vs. vehicle control.

Since lung tumor cells have higher expression of 27HC-generating CYP27A1 (Figure 1A), it is plausible that endogenous 27HC production affects ERβ (+) lung cancer cell proliferation. To examine the involvement of endogenous 27HC production in H1395 cell proliferation, we introduced shRNA against *CYP27A1* into H1395 cells and generated clones A3 and D2. The shRNA decreased the expression of *CYP27A1* significantly in two clones, A3 and D2 (Figure 4A); however, the cell proliferation was not altered in the presence of control or shRNA against *CYP27A1* (Figure 4B). In a similar fashion, siRNA mediated suppression of *CYP7B1*, #32 and #33, significantly decreased *CYP7B1* mRNA levels in H1395 cells (Figure 4C) but did not alter cell proliferation relative to control siRNA cells (Figure 4D). We further confirmed our findings using clotrimazole, an inhibitor of P450 enzymes to which both CYP27A1 and CYP7B1 belong, and the treatment of H1395 cells at 1 μM did not alter cell proliferation (Figure 4E). These results indicate that although lung cancer cells have high 27HC-generating enzyme expression, endogenous 27HC is not the major effector of ERβ (+) lung cancer cell proliferation.

### 27HC promotes cell proliferation through the PI3K-Akt pathway

**Figure 5:**
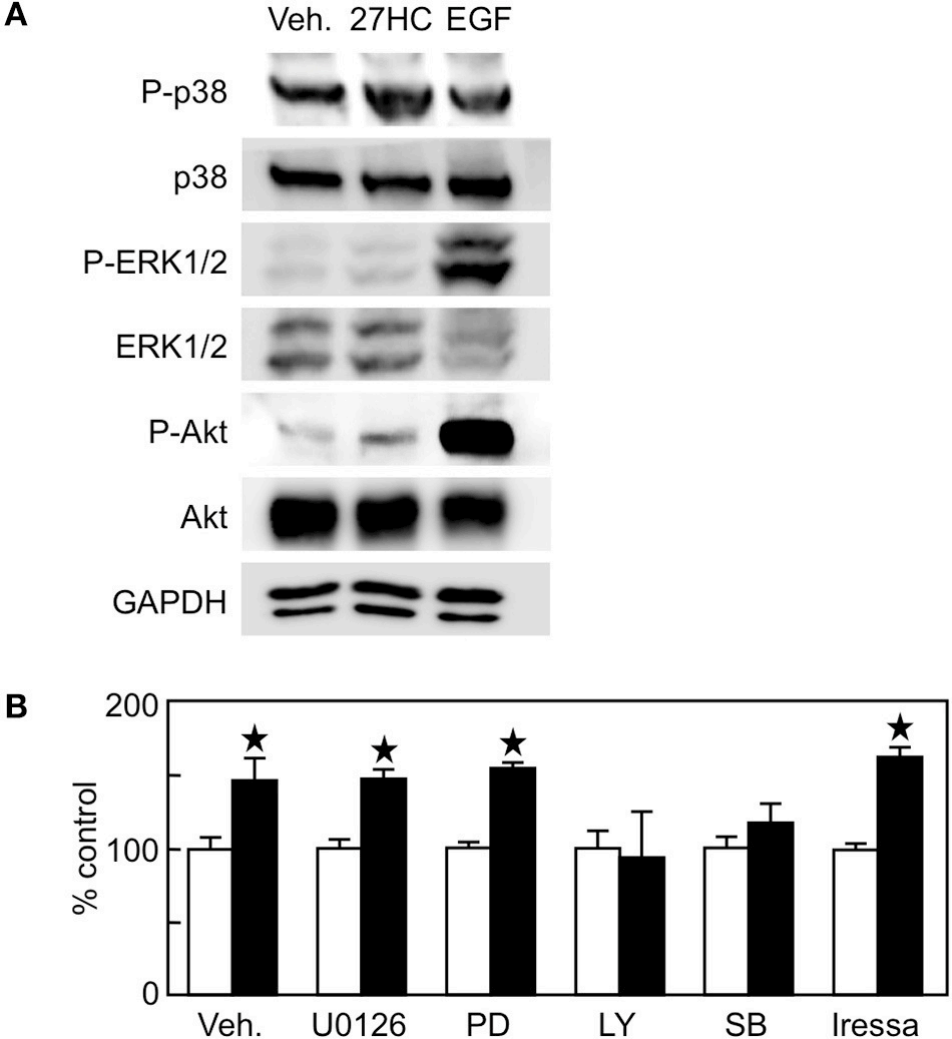
The effect by 27HC on cell proliferation is mediated by PI3K-Akt signaling pathway. **(A)** Immunoblot of phospho-p38 MAPK, total p38 MAPK, phospho-ERK1/2, total ERK1/2, phospho-Akt, and total Akt proteins. H1395 cells were treated with vehicle (Veh.), 10 μM 27HC, or 10 ng/ml EGF for 30 min, and protein abundance of phosphorylated or total proteins in the cell lysates were detected. **(B)** H1395 cell proliferation with kinase inhibitors. U0126 (1 μM), PD0325901 (1 μM), LY294002 (5 μM), SB203580 (1 μM), and iressa (1 μM) were treated 1 h before the 27HC treatment throughout the experiment (*n* = 4). Open bars, vehicle treatments; closed bars, 27HC (1 μM) treatments. ^*^p < 0.05 vs. vehicle control.

In addition to its action as a transcription factor, ER has an action as an extranuclear signaling molecule involving various kinase pathways such as PI3K-Akt and MEK-MAPK pathways. In non-small cell lung cancer, ER activation leads to the induction of the p44/p42 MAPK signaling pathway, which is considered to be an extranuclear action of ER (14). In addition, ER interacts with EGF receptor and activate tyrosine kinase signaling in breast cancer (15), and EGF, but not IGF or VEGF, promotes H1395 cell proliferation (Supplemental Figure 3A). To examine whether 27HC is capable of eliciting extranuclear effects, we performed immunoblot analysis using H1395 cell extracts. EGF stimulated the phosphorylation of p38 MAPK, ERK1/2, and Akt in H1395 cells (Figure 5A). The treatment of 27HC also phosphorylated p38 MAPK and Akt, but not ERK1/2. Next, we performed cell proliferation assays in combination with kinase inhibitors. These inhibitors suppressed basal cell growth (Supplemental Figure 3C). The effect by 27HC was not inhibited by MEK/MAPK inhibitors PD0325901 or U0126 (Figure 5B). In contrast, the effect by 27HC was suppressed in the presence of the PI3K inhibitor LY294002. Interestingly, another p38MAPK inhibitor SB203580, which also inhibits Akt, suppressed the effect of 27HC. To examine the involvement of the EGFR signaling pathway in the ERβ-mediated 27HC effect on cell proliferation, EGFR inhibitor Irressa was also used. Iressa at 1 μM, which completely blocked the cell proliferation induced by EGF (Supplemental Figure 3B), did not alter the effects of 27HC (Figure 5B). These results suggest that 27HC promotes H1395 cell proliferation through the activation of PI3K-Akt signaling pathway in ERβ (+) lung cancer cells.

## Discussion

Although it is still controversial whether plasma cholesterol levels correlate with lung cancer risks, and also whether higher body mass index (BMI) is protective or harmful to lung cancer depends on cancer types, sex, and treatments (30–32), statins that lower cholesterol levels reduce the risk and mortality of lung cancer in human (33, 34), suggesting that lowering cholesterol levels, which also lowers 27HC levels, has a beneficial effect against lung cancer. Nevertheless, the precise mechanism of how cholesterol affects lung cancer pathogenesis, especially in relation with estrogen receptor status, still remains unknown. In addition, among many risk factors that may contribute to lung cancer incidence or mortality, endocrine factors including estrogen are critical modulators of lung cancer development and progression, and there is accumulating evidence that ERβ expression in conjunction with aromatase expression predicts survival in non-small cell lung cancer both in men and women (35, 36). Despite the poorly understood role of cholesterol in lung cancer pathogenesis, lung tissue has abundant levels of 27HC over a range of concentrations relevant to the modulation of ER function (18, 37). Additionally, protein expression of CYP27A1 is higher in lung tissues from chronic obstructive pulmonary disease patients than normal lung tissues (38). With the gene expression results showing that 27HC production and ERβ expression is higher in lung tumor cells than in normal lung cells (Figure 1), we determined if 27HC modifies lung cancer cell proliferation, and found that 27HC promotes cell proliferation of lung cancer cells that express ERβ. Although some reports suggested that 27HC is a weak agonist for LXR (39), 27HC does not activate LXR in the vascular system and in liver (18). The effect of 27HC on H1395 cells was ERβ-specific, and other nuclear receptor ligands tested did not show proliferation-inducing effects (Figure 3). The effect of 27HC was observed not only in H1395 cells, but also in LLC cells that are transfected with ERβ (Supplemental Figure 1), indicating the effect of 27HC is not H1395 cell-specific. Recently, a report indicated that 27HC acts as a negative modulator of ERβ (40). In our study, both 27HC and E_2_ promoted H1395 cell proliferation. Considering estrogen always activates ER, 27HC likely has an ERβ-mediated, pro-estrogenic function in lung cancer cells. Since 27HC shows both agonistic and antagonistic effects on ERα function depending on target organs in our previous studies (16), it is possible that 27HC may also function as both an agonist and antagonist for ERβ in a cell/tissue type specific manner.

Interestingly, although the expression of *CYP27A1* is higher in lung cancer cells than in normal lung cells (Figure 1), endogenously produced 27HC did not alter lung cancer cell proliferation in this study (Figure 4). The production of 27HC in lung cancer cells was not be determined in this study, however, there is accumulating evidence that *CYP27A1* mRNA expression correlates with its protein expression and enzymatic activity, and also that CYP27A1 expression correlates with the levels of its product 27HC in various types of cells and also *in vivo* (41–43). In addition, to our knowledge, there is no other enzyme than CYP27A1 known to endogenously produce 27HC in mammals. Thus, it is reasonable to assume that higher CYP27A1 expression reflects higher 27HC production in lung cancer cells, and also there are reports showing effective *CYP27A1* knockdown and consequent decrease of its protein expression (44, 45). In breast cancer, resident macrophages are important for generating and providing 27HC to the microenvironment (21). In addition to the key involvement of ERβ in lung tumor cells, ER activation by 27HC in surrounding stromal cells may also contribute importantly to oncogenesis, such as the regulation of host immunity by estrogen, which is observed in breast tumor models (46). Other possibilities include that the experimental condition in this study was not suitable for detecting the subtle differences caused by endogenously produced 27HC, and also that there are some effects by endogenous 27HC other than those that we examined. Further study will examine the role of endogenously produced 27HC in lung cancer cells and the major source of 27HC involved in lung cancer cell proliferation.

In this study, we found that the effect of 27HC is stronger than that of E_2_, even with superphysiological doses of E_2_ (Figure 2 and data not shown). Mechanistic studies indicated that the effect of 27HC is mediated not by EGFR or MAPK, but by the PI3K-Akt signaling pathway (Figure 5). We could not find evidence that 27HC acts on pathways other than ER; however, there is still a possibility that 27HC acts on unknown pathways, if any, in addition to the ER signaling pathway. The PI3K-Akt signaling pathway is involved in lung cancer progression (47), and 27HC acts on the cardiovascular system through the activation of PI3K-Akt signaling pathway (48). Further investigation on how 27HC affects the PI3K-Akt signaling pathway are necessary to clarify the role of PI3K-Akt signaling on lung cancer progression.

The novel nature of 27HC modulation of ERβ that we show in this study has revealed a theoretical concept that links cholesterol metabolism with lung cancer progression and treatment failure. Such studies will further our understanding of lung tumor pathogenesis and will further indicate the efficacy of 27HC-directed therapeutic interventions for the treatment of this deadly disease.

## Author contributions

SH, TI, and MU designed the experiments. JM provided cell lines. SH, TI, W-RL, TK, CC, NK, FZ, and AA performed the experiments and acquired the data. SH, TI, MS, YZ, JH, JM, and MU analyzed and interpreted the results, and MU wrote the manuscript.

### Conflict of interest statement

The authors declare that the research was conducted in the absence of any commercial or financial relationships that could be construed as a potential conflict of interest.
