# Supplementary material for: Estrogen Receptor Beta-Mediated Modulation of Lung Cancer Cell Proliferation by 27-Hydroxycholesterol
Source: Front Endocrinol (Lausanne). 2018 Aug 23;9:470. doi: 10.3389/fendo.2018.00470 (PMC6116707; doi:10.3389/fendo.2018.00470)
Supplement: Supplementary file 1 [file Data_Sheet_1.pdf]

Supplementary Material

Estrogen Receptor Beta-mediated Modulation of Lung Cancer Cell Proliferation by 27-Hydroxycholesterol

Shiro Hiramitsu, Tomonori Ishikawa, Wan-Ru Lee, Tamor Khan, Christine Crumbley, Nimra Khwaja, Faezeh Zamanian, Arvand Asghari, Mehmet Sen, Yang Zhang, John R Hawse, John D Minna, and Michihisa Umetani

*** Correspondence:** Michihisa Umetani: mumetani@uh.edu

1 Supplementary Table

Gene Analyzed	Accession Number	QPCR Primer Sequences
ERα	NM_000125	5’ agagaagtattcaaggacataacgactatat 3’<br>5’ tcttcctcctgtttttatcaatgg 3’
ERβ	NM_001437	5’ aagttggccgacaaggagtt 3’<br>5’ acaggctgagctccacaaag 3’
CYP27A1	NM_000784	5’ tgcggcaagagggaaagta 3’<br>5’ cggtgctccttccatagct 3’
CYP7B1	NM_004820	5’ tgtgtgcgaaagggagacttggta 3’<br>5’ agtttctcctgtgagcacagtttg 3’
Cyclophilin	NM_000942	5’ ggagatggcacaggaggaa 3’<br>5’ gcccgtagtgcttcagttt 3’

Supplementary Table 1. Primer sequences used for RT-qPCR.

2 Supplementary Figures

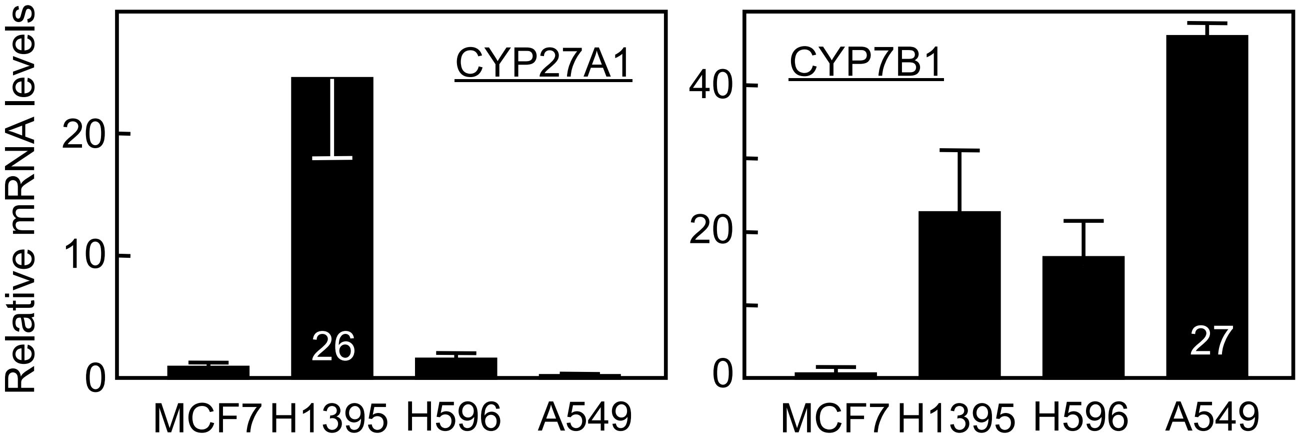

Supplementary Figure 1. Gene expression of *CYP27A1* and *CYP7B1* in human cancer cells. qRT-PCR analysis of MCF7, H1395, H596, and A549 cancer cells (n=6-7). Cycle time of the highest expressing group for each gene is indicated inside its corresponding bar.

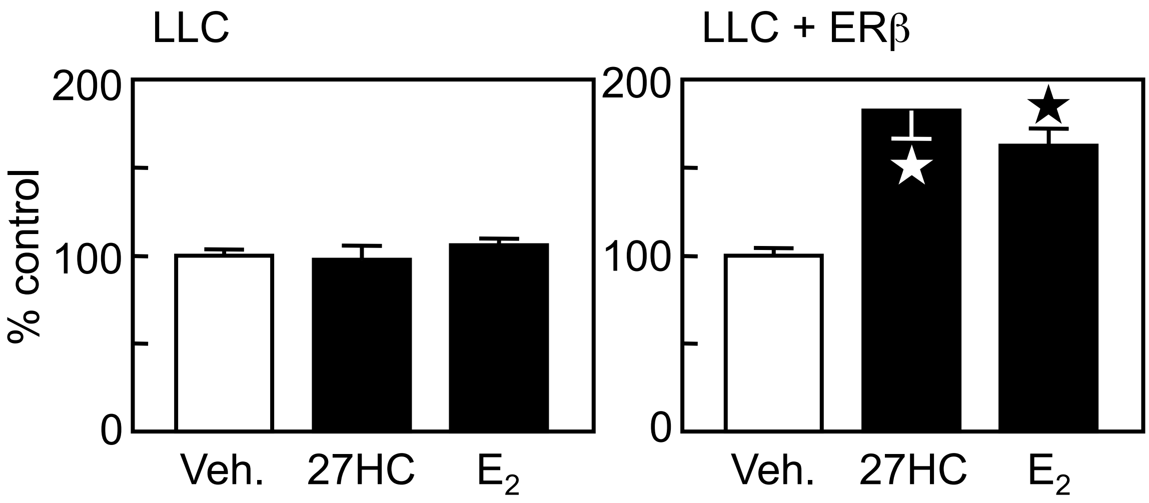

Supplementary Figure 2. Effect of 27HC on lung cancer cell proliferation is ERβ-dependent. Cell proliferation assay in LLC and LLC overexpressing ERβ. Cells were treated with vehicle, 27HC (1 μM), or E_2 (10nM) for 24 hrs (n=4). *p<0.05 vs. vehicle control.

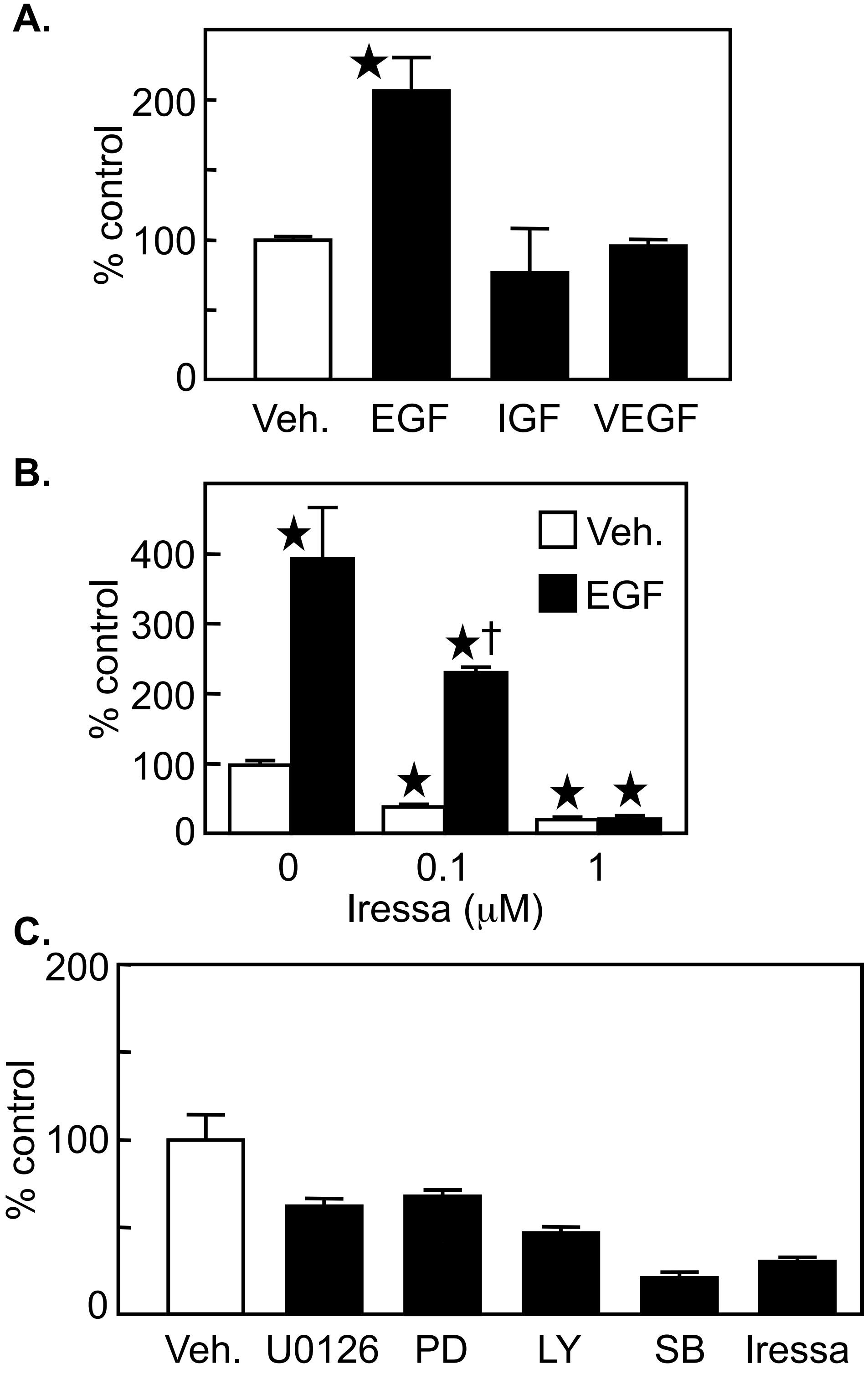

Supplementary Figure 3. Effect of kinase inhibitors on H1395 cell proliferation. **A**. Cell proliferation assay of H1395 cells with growth factors (n=4). **B**. H1395 cells were treated with Iressa 1 hr before the EGF treatment (10 ng/ml) throughout the experiment (n=4). *$p<0.05$ vs. vehicle without Iressa, †$p<0.05$ vs. vehicle with the same dose of Iressa. **C**. U0126 (1 μM), PD0325901 (1 μM), LY294002 (5 μM), SB203580 (1 μM), and Iressa (1 μM) were added for 25 hrs (n=4).
